# Metabolic Profiling Reveals Diagnostic Biomarkers for Distinguishing Myocarditis From Acute Myocardial Infarction

**DOI:** 10.1155/cdr/6292099

**Published:** 2025-04-16

**Authors:** Yuting Chen, Xiu Liu, Chengying Hong, Shunyao Xu, Linling He, Zhenmi Liu, Huaisheng Chen, Yaowang Lin

**Affiliations:** ^1^Department of Critical Medicine, Shenzhen People's Hospital, First Affiliated Hospital of Southern University of Science and Technology, Second Clinical Medicine College of Jinan University, Shenzhen, China; ^2^Department of Cardiology, Shenzhen People's Hospital, First Affiliated Hospital of Southern University of Science and Technology, Second Clinical Medicine College of Jinan University, Cardiovascular Minimally Invasive Medical Engineering Technology Research and Development Center, Shenzhen Key Medical Discipline (SZXK003), Shenzhen, China

**Keywords:** 11-*cis*-retinol, acute myocardial infarction, metabolic reprogramming, myocarditis, retinol metabolism

## Abstract

**Background:** Distinguishing between myocarditis (MC) and acute myocardial infarction (AMI) in the early stages is crucial due to their similar symptoms yet vastly different treatment protocols. This study seeks to utilize metabolomics techniques to differentiate between MC and AMI.

**Methods:** Plasma samples from 15 patients with MC and 12 patients with AMI were collected. Metabolic profiles of plasma from the two groups of patients were obtained using ultra-high performance liquid chromatography–mass spectrometry (UHPLC-MS), identifying metabolites with significant differences.

**Results:** We identified 30 significantly different metabolites in both diseases. In patients with MC, 17 metabolites were upregulated, including 5-hydroxy-L-tryptophan and LysoPC (18:2(9Z,12Z)), while 13 metabolites were downregulated, such as 11-*cis*-retinol, L-glutamate, and hydroxynicotinic acid. KEGG enrichment analysis revealed that the altered metabolites were enriched in tryptophan metabolism, linoleic acid metabolism, primary bile acid biosynthesis, nitrogen metabolism, and retinol metabolism. Biomarker analysis via receiver-operating characteristic curves highlighted 11-*cis*-retinol as the predominant biomarker, with an AUC value of 0.917.

**Conclusions:** In conclusion, patients experiencing AMI and MC undergo significant metabolic reprogramming. Metabolites exhibiting abnormal expression in peripheral blood hold diagnostic value for distinguishing between AMI and MC in clinical settings. 11-*cis*-retinol proved to be the pivotal biomarker for AMI, potentially aiding in the development of a robust predictive model for distinguishing between MC and AMI in clinical settings.

## 1. Introduction

Myocarditis (MC), an inflammatory disease, arises from direct or indirect damage to myocardial cells induced by viral infections, autoimmune diseases, drug reactions, toxin exposure, and other factors [1, 2]. It primarily results from the degeneration and necrosis of myocardial cells, the infiltration of inflammatory cells in the interstitium, and the exudation of fibers, causing various degrees of cardiac dysfunction [3]. Endomyocardial biopsy serves as the gold standard for diagnosis [4], although clinical diagnosis often relies on cardiac magnetic resonance imaging (CMRI) analysis [5]. For young athletes, it stands as the third most common reason for cardiovascular death (6%), trailing behind coronary artery anomalies (17%) and hypertrophic cardiomyopathy (36%) [6]. Early symptoms of MC comprise respiratory and gastrointestinal infections, along with chest pain and, in severe cases, heart failure [7]. In the early stages, MC lacks a specific diagnostic index, and its symptoms, electrocardiographic changes, and elevated cardiac enzymes closely resemble those of acute myocardial infarction (AMI), posing a risk of misdiagnosis [8, 9]. Hence, there is an urgent need to identify a noninvasive/minimally invasive, rapid, and precise biomarker for the differential diagnosis of MC and AMI.

Metabolites, arising from a combination of genetic material and environmental influences, make metabolomics the research method most closely aligned with the phenotype. Metabolomics, along with pathway analysis, can unveil disease-specific biomarkers, thereby facilitating clinical diagnosis and disease classification [10, 11]. Metabolomics has unveiled numerous metabolic markers in cardiovascular disease research closely linked to disease progression, encompassing glucose metabolism [12], fatty acid pathways [13], amino acid pathways [14], and beyond. Nevertheless, potential biomarkers for early detection of MC and AMI in patients based on metabolomics remain unexplored.

In this study, 27 patients who underwent coronary angiography (CAG) between May 2022 and May 2023 in our hospital were assigned to two groups: patients who were diagnosed with AMI and patients who were diagnosed with MC. The plasma samples from the included patients were collected for untargeted metabolomic analysis. The main objective of this study was to identify metabolites with differential expression in the peripheral blood of patients with AMI or MC and to screen for metabolites with the potential to assist in distinguishing between the two conditions.

## 2. Materials and Methods

### 2.1. Design of the Study and Recruitment of Participants

Twenty-seven patients who underwent CAG between May 2022 and May 2023 were assigned to two groups in this prospective observational study: patients who were diagnosed with AMI (AMI group, *n* = 12) and patients who were diagnosed with MC (MC group, *n* = 15). The diagnostic criteria for viral MC are CAG to exclude coronary stenosis, along with endomyocardial biopsy and cardiac enhancement nuclear magnetic resonance to confirm acute MC. All the individuals involved agreed to provide written informed consent. The study approach was approved by the hospital's institutional review board.

### 2.2. Ultra-Performance Liquid Chromatography (UPLC)/Mass Spectrometry (MS) Sample Preparation

Blood was collected immediately after diagnosis to identify different metabolites in the early stages of the two diseases. A patient venous blood sample was stored in an ethylenediaminetetraacetic acid (EDTA) anticoagulant tube. Samples were centrifuged at 1600 g for 10 min to isolate the plasma, which was subsequently stored at −80°C until analysis. Before analysis, the samples were thawed on ice and mixed with precooled 50% methanol (1:6), vortexed for 1 min, and incubated at room temperature for 10 min. They were stored at −20°C overnight and then centrifuged at 4000 g for 20 min. Samples were then kept at −80°C until analysis by LC-MS.

The analysis of all specimens followed a set sequence using an LC-MS system. The separation process was carried out utilizing an UPLC system fitted with an ACQUITY UPLC T3 column (100 × 2.1 mm, 1.8 *μ*m). Metabolites were detected using a high-resolution Triple TOF 5600+ tandem mass spectrometer in both positive and negative ion modes. Acquisition of data utilized an approach known as information-dependent acquisition (IDA) mode, covering a TOF mass range from 60 to 1200 Da.

### 2.3. Data Processing and Analysis

Various processes including the extraction of peaks, grouping of peaks, correction of retention times, grouping of secondary peaks, and identification of isotopes and adducts were carried out on the acquired MS data. LC-MS raw data underwent conversion to mzXML format followed by processing in the R software package. Metabolite identification was conducted using the Human Metabolome Database (HMDB, https://hmdb.ca/) based on retention time and mass-to-charge ratio data, with a mass accuracy threshold of ≤ 10 ppm. KEGG metabolic pathway analysis was performed using the MetaboAnalyst 3.0 platform (http://www.metaboanalyst.ca). Metabolites with a mass accuracy of ≤ 10 ppm were mapped to the KEGG database, and pathway significance was assessed using a hypergeometric test with a false discovery rate (FDR) threshold of < 0.05. Enrichment results were visualized based on pathway impact scores derived from topology analysis, with *p* values adjusted using the Benjamini–Hochberg method.

### 2.4. Statistics Analysis

Statistical analyses were performed using SPSS 22.0 and GraphPad Prism 8.0.1, with mean ± standard deviation (±SD) used for normally distributed measures. Group comparisons were carried out using *t*-test or *t*′-test. Non-normally distributed quantitative data were presented as median and interquartile range. Differences between groups were evaluated through Student's test or the Kruskal–Wallis test for continuous variables and chi-square test or Fisher's exact test (as appropriate) for categorical variables. Metabolic pathway and biomarker studies were performed using the free online software MetaboAnalyst Version 5.0 (Wishart Research Group, Canada). The area under the curve (AUC) of differential metabolites was analysed using the receiver operating characteristic (ROC) curve. *p* values < 0.05 were considered statistically significant.

## 3. Results

### 3.1. Clinical Characteristics of MC and AMI Patients

Twenty-seven patients were recruited in this study, including 15 MC patients and 12 AMI patients. Baseline characteristics of the MC and AMI groups are shown through Table 1. We found that the MC group was younger and had lower LDL and higher CPR (*p* < 0.05). Gender, body mass index (BMI), vital signs, and other laboratory indicators were similar between the two groups.

### 3.2. Serum Metabolic Profiling in MC and AMI Patients

**Figure 1:**
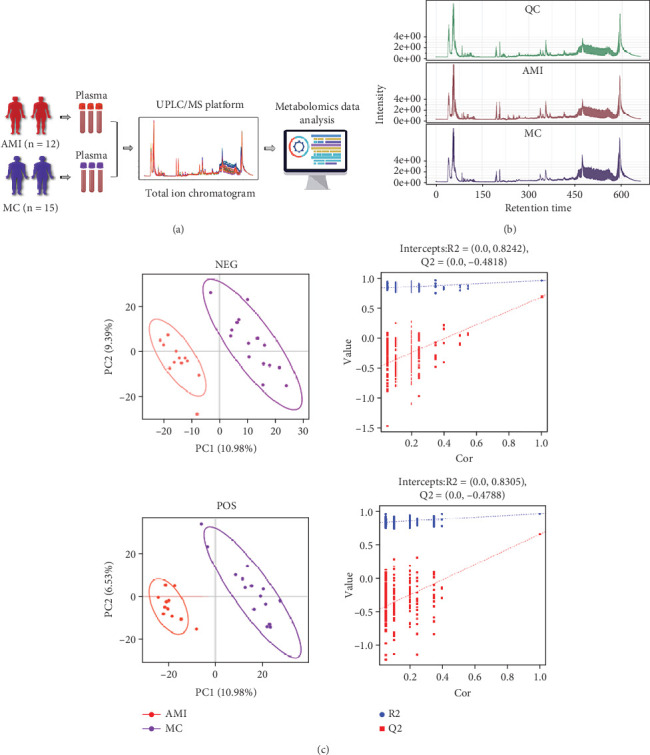
Differential metabolites screening between myocarditis (MC) and AMI patients. (a) Schematic diagram of the workflow for the analysis of the metabolome. (b) Typical total ion current chromatogram from two groups in UPLC/MS. (c) PLS-DA plot of two groups and validation of the PLS-DA model.

Chromatographic peaks of the total ion chromatogram were analysed under UPLC conditions and MS conditions (Figure 1a,b). Multivariate statistical analysis through the use of partial least squares–discriminant analysis (PLS-DA) was implemented for the identification of varied metabolites. In PLS-DA, the sera of the MC and AMI groups clearly separated the blocks (Figure 1c). Furthermore, validation through R2 and Q2 results indicated the robustness and reliability of the PLS-DA model without signs of overfitting.

### 3.3. Comparison of Characteristic Metabolites in the Plasma of MC Patients and AMI Patients

**Figure 2:**
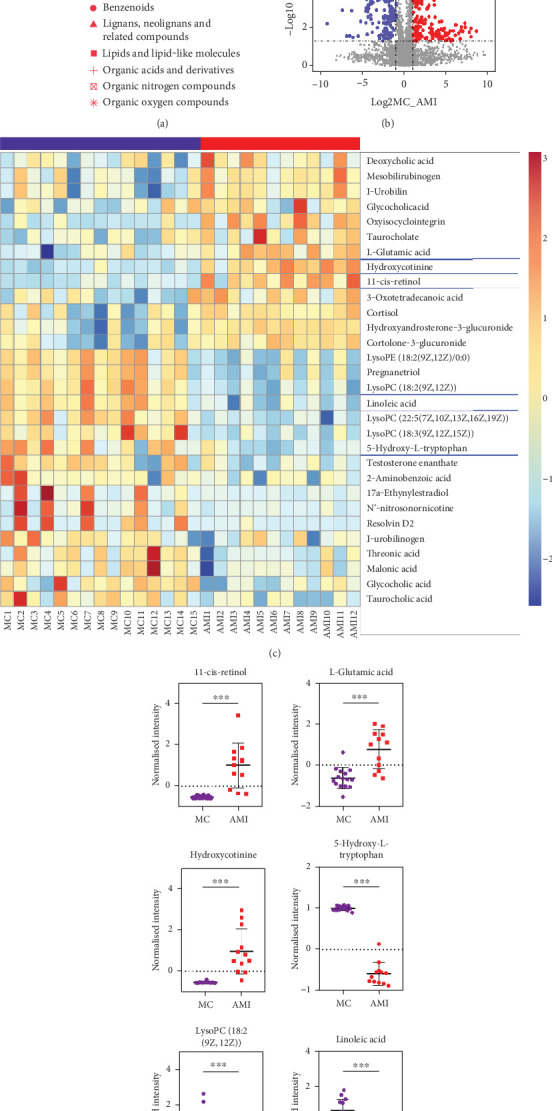
Metabolite compositions. (a, b) Volcano plot of different metabolites and different ion between the myocarditis and AMI groups. (c) Heat map analysis of metabolite compositions between the myocarditis and AMI groups. (d) Two-tailed *t*-tests for quantitative levels of differential metabolites.

Subsequently, we found that the differential metabolites in the plasma of MC and AMI patients were mainly lipids and lipid-like molecules, organic oxygen compounds, organic acids and derivatives, organic cyclic compounds, and benzene-like compounds. Compared to AMI, MC patients' plasma showed a total of 173 upregulated metabolites and 131 downregulated metabolites (Figure 2a,b). We further analysed the metabolites and found that 30 metabolites were significantly different (VIP > 1, *p* < 0.05, *R* > 2, or *R* < 0.5), of which 17 metabolites were upregulated (VMC/AMI, *R* > 2), such as linoleic acid, 5-hydroxy-L-tryptophan, and LysoPC (18:2(9Z,12Z)); 13 metabolites were downregulated (VMC/AMI, *R* < 0.5) such as 11-*cis*-retinol, L-glutamic acid, and hydroxycotinine (Figure 2c,d). These findings suggest that metabolites such as 11-*cis*-retinol may have the potential to assist in distinguishing between AMI and MC.

### 3.4. Metabolic Pathways and Functional Analysis

**Figure 3:**
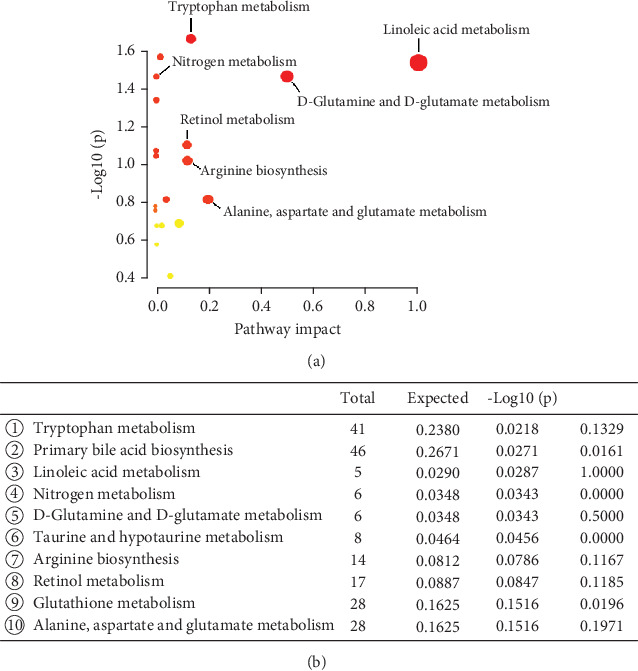
(a, b) The metabolic pathways identified using MetaboAnalyst 5.0 software.

**Figure 4:**
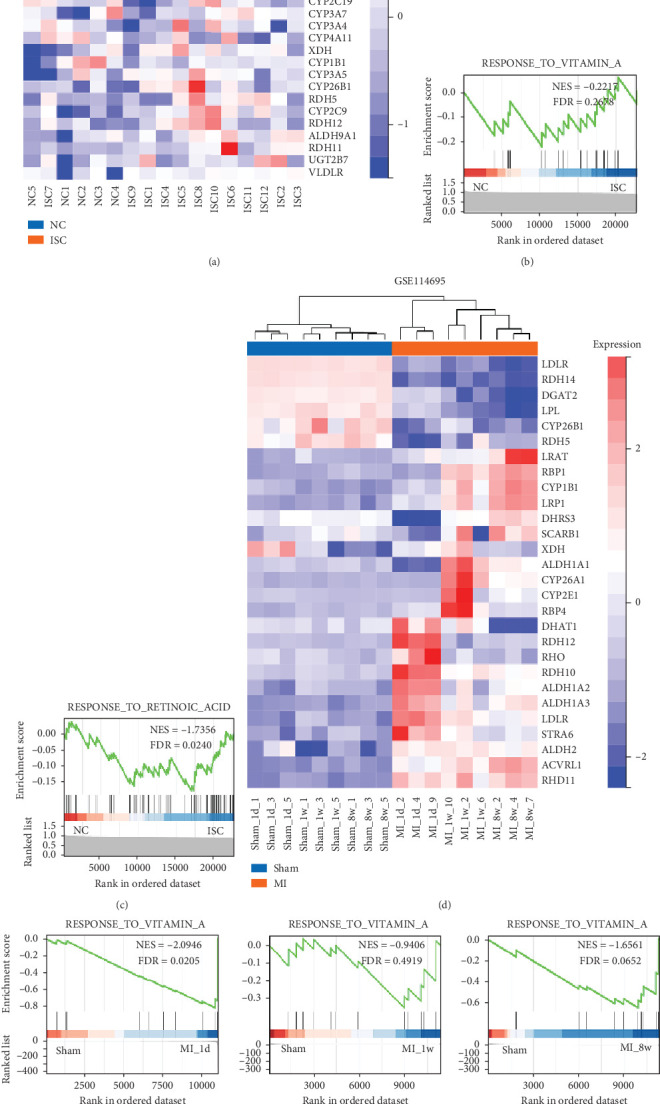
Gene set enrichment analysis of significant differentially expressed mRNAs with normalized enrichment score (NES) and FDR *Q*-value. (a, d) Heat map depicting changes in the expression of genes involved in GSE42955 and GSE 114695. (b, c, e, f) The results of “VITAMIN_A” and “RETINOIC_ACID” gene set enrichment analysis (GSEA).

KEGG metabolic libraries analysis showed that the altered metabolites were enriched in tryptophan metabolism, primary bile acid biosynthesis, linoleic acid metabolism, nitrogen metabolism, retinol metabolism, and other processes (Figure 3a,b). To further investigate metabolite changes related to MC and AMI, RNA-seq data from the cardiac tissue for 12 ischemic cardiomyopathy patients and four normal cardiac tissues were downloaded (Accession Number GSE42955). Gene set enrichment analysis (GSEA) showed that “VITAMIN_A” and “RETINOIC_ACID” were remarkably enriched in the AMI group, with enrichment scores of −0.2117 (FDR = 0.2678) and −17.356 (FDR = 0.0240), respectively (Figures 4a, 4b, and 4c). This suggests an increased enrichment of retinol metabolism–related genes in the ischemic cardiomyopathy group compared with the normal group. We also downloaded cardiac tissue RNA-seq transcriptomics data from the GEO database (Accession Number GSE114695) for 1 day (1D), 1 week (1W), and 8 weeks (8W) after MI and the normal group of mice. We found that “VITAMIN_A” and “RETINOIC_ACID” were also remarkably enriched in the 1D MI group, 1W MI group, and 8 W MI group, respectively (Figures 4d, 4e, and 4f). Therefore, these findings strongly suggest a reprogramming of retinol metabolism in the myocardial tissue of AMI patients, resulting in the release of 11-*cis*-retinol into the bloodstream. The high levels of retinoic acid in patient plasma serve as a crucial marker for AMI and can be utilized to differentiate it from MC.

### 3.5. Metabolic Biomarker Evaluation

**Figure 5:**
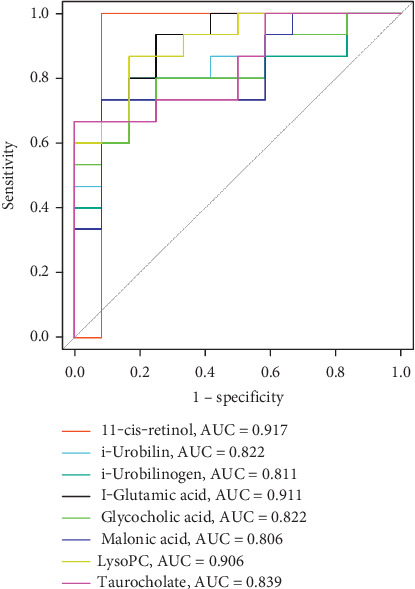
Univariate subject operating characteristic curves for each biomarker identification.

11-*cis*-retinol received the highest AUC (0.917) in univariate analysis for biomarker identification with ROC curves, suggesting it can be used as a metabolic biomarker to distinguish MC from AMI (Figure 5).

## 4. Discussion

In this study, we successfully extracted the metabolic characteristics of peripheral blood from patients with MC and AMI using metabolomics techniques. Moreover, we discovered that AMI patients' myocardial tissue undergoes a reprogramming of retinol metabolism, highlighting the diagnostic potential of 11-*cis*-retinol levels in peripheral blood for distinguishing between MC and AMI.

MC is the degeneration, necrosis, and concomitant inflammatory response of cardiomyocytes due to direct attack by infectious agents, abnormal activation of the immune system, and adverse reactions to drugs or vaccines [2]. AMI is mainly caused by vascular occlusion due to thrombus formation on the basis of coronary atherosclerosis, which triggers damage or death of cardiomyocytes due to hypoxia [15]. These two diseases are difficult to differentiate in the early stages of their onset, mainly due to the extreme similarity of their clinical symptoms, both of which may present with changes such as chest pain, changes in electrocardiogram (ECG), and elevated levels of cardiac biomarkers. However, there are significant differences in the treatment and prognosis of these two diseases, which highlights the importance of accurate and timely diagnosis. Currently, MC is mainly diagnosed clinically by performing PCI to rule out AMI or by perfecting CMRI [16], and there is no easy, rapid, and noninvasive method of differential diagnosis.

Metabolomics can be used to identify potential markers of disease, reveal pathological mechanisms of disease, and identify targets for drug action [17]. It has been shown that myocardial infarction is associated with metabolic abnormalities. In a large meta-analysis of six international cohorts, researchers identified 10 new and 46 known metabolites associated with myocardial infarction events through metabolite assays and quantification of serum samples from the participants, and these metabolic profiles may help identify high-risk individuals before onset [18]. In addition, Hoshi et al. found that inhibition of the L-Trp-Kyn metabolic pathway increased survival and reduced viral load in mice with acute viral MC by examining metabolite changes in mouse serum [19]. Therefore, we speculate that there may be some differential metabolites in the plasma of patients with MC and AMI, which may provide new ideas and methods for early differential diagnosis.

In this study, metabolomics was used for the first time to analyse differentially altered metabolites in the early stages of MC and AMI pathogenesis. By comparing plasma samples from MC and AMI patients, 30 significantly altered metabolites were found in patients with AMI. We observed that linoleic acid, 5-hydroxy-L-tryptophan, and LysoPC (18:2(9Z,12Z)) were specifically elevated in the peripheral blood of patients with MC, suggesting their potential as MC-specific biomarkers. In contrast, compared to MC patients, the levels of 11-*cis*-retinol, L-glutamic acid, and hydroxynicotinine were significantly increased in the peripheral blood of AMI patients, likely reflecting metabolic reprogramming in cardiomyocytes under hypoxic conditions.

In addition, we downloaded RNA-seq transcriptomics data from the GEO database for patients with ischemic cardiomyopathy and normal cardiac tissues and found that the ischemic cardiomyopathy group showed an increased enrichment of genes related to retinol metabolism compared with the normal group. Meanwhile, we also downloaded cardiac tissue RNA-seq transcriptomic data from the GEO database for mice at 1D, 1W, and 8W and the normal group after MI, and we found that retinol metabolism pathways were significantly increased in myocardial infarction mice. Retinol metabolism is a complex biochemical process that involves a variety of enzymes and receptors and plays an important role in the visual cycle, cell differentiation, cell proliferation, and the development of a variety of diseases [20]. Retinol-binding protein 4 (RBP4) is a serum protein primarily responsible for retinol transport [21]. Lambadiari et al. found that RBP4 serum levels were notably elevated among individuals diagnosed with coronary artery disease in comparison to those without the condition [22]. Meanwhile, Zhang et al. found that in AMI mouse models and ischemic hypoxia–induced cardiomyocyte injury models, increased expression of RBP4 activated the NLRP3 inflammasome, promoted the cleavage of caspase-1 precursor, and subsequently induced GSDMD-dependent cleavage [23]. In addition, Luo et al. found that the retinol metabolic pathway was a better predictor of early ventricular fibrillation in ST-segment elevation myocardial infarction (STEMI) by collecting plasma from patients with STEMI and performing metabolite assays [24]. From this, we can see that retinol metabolism plays an important role in the pathogenesis of AMI, which is consistent with our findings. Therefore, we believe that retinol metabolism is more different in the two diseases and has some clinical significance.

The AUC for 11-*cis*-retinol was calculated to be 0.917 by ROC curve analysis, which indicates that 11-*cis*-retinol is the most discriminating biomarker. Wu et al. found that retinol excess and all-trans retinoic acid deficiency in mouse cardiac tissues increased lipid deposition and free fatty acid uptake, promoted lipotoxicity in the heart, and concomitantly inhibited glutathione peroxidase 4 (GPX4) and iron death inhibitory protein 1 (FSP1) expression [25]. These findings also provide additional evidence for our finding that 11-*cis*-retinol can be a potential target for discriminating early MC from AMI.

Despite the progress made in this study, there are some limitations. First, the sample size was relatively small, and further expansion of the sample size is needed to illustrate the generality of the results. Second, no external validation experiments were conducted as a way to increase the credibility of the results. In addition, due to the limitations of the study design, we were unable to exclude the influence of other potential confounding factors on metabolite levels, such as dietary and lifestyle factors.

## 5. Conclusion

In this study, metabolomics technology unveiled metabolic distinctions between MC and AMI, successfully pinpointing potential biomarkers for early identification of both conditions. This discovery holds significant clinical implications, facilitating prompt detection of these diseases at their onset. Early and precise intervention prompted by such identification can enhance patient prognosis significantly.

## Figures and Tables

**Table 1:** Clinical characteristics of the two groups.

	**MC (** **n** = 15**)**	**AMI (** **n** = 12**)**	**p** ** value**
Characteristics			
Age (years)	28.38 ± 10.26	42.33 ± 12.79	**0.006**
Males	11 (73.33%)	9 (75.00%)	0.921
Body mass index (kg/cm^2^)	23.62 ± 3.23	24.08 ± 3.81	0.742
SBP (mmHg)	112.50 ± 15.59	127.20 ± 24.42	0.079
HR (bpm)	74.38 ± 13.81	79.00 ± 12.86	0.622
Laboratory data			
White blood cells (×10^9^/L)	9.88 ± 2.75	11.3 ± 2.68	0.194
NLR (%)	5.44 ± 1.93	4.81 ± 2.19	0.443
Hemoglobin (g/L)	141.80 ± 11.56	145.30 ± 12.11	0.453
Platelets (×10^9^/L)	236.20 ± 43.45	268.50 ± 44.67	0.073
Albumin (g/L)	42.21 ± 4.25	42.55 ± 4.57	0.847
HbA1c (%)	5.44 ± 0.20	5.70 ± 0.36	0.227
Potassium (mmol/L)	3.78 ± 0.52	3.89 ± 0.44	0.560
Uric acid (*μ*mol/L)	354.50 ± 142.30	395.70 ± 78.95	0.371
Creatinine (*μ*mol/L)	78.26 ± 13.22	80.85 ± 17.06	0.669
HDL-C (mmol/L)	0.91 ± 0.32	0.90 ± 0.20	0.937
LDL-C (mmol/L)	2.174 ± 0.72	2.92 ± 0.57	**0.004**
C-reactive protein (mg/L)	49.48 ± 28.67	10.27 ± 6.07	**0.003**
Procalcitonin (ng/mL)	0.09 ± 0.04	0.06 ± 0.04	0.280
Lactate (mmol/L)	2.82 ± 1.0	3.51 ± 1.32	0.160
CK-MB (U/L)	40.70 ± 21.42	51.84 ± 29.12	0.348
cTnI (ng/mL)	5.67 ± 3.53	6.28 ± 4.42	0.740
D-dimer (mg/L)	426.30 ± 213.10	301.70 ± 74.09	0.086

*Note:* Bold data indicates a statistically significant difference at *p* < 0.05.

## Data Availability

Due to restrictions on privacy, sharing the datasets provided in this paper is not feasible. Inquiries regarding the datasets should be directed to the corresponding author.
